# Optimal Flow and Pressure Management in Machine Perfusion Systems for Organ Preservation

**DOI:** 10.1007/s10439-012-0601-9

**Published:** 2012-06-06

**Authors:** Ivo C. J. H. Post, Marcel C. Dirkes, Michal Heger, Rick Bezemer, Johan van ‘t Leven, Thomas M. van Gulik

**Affiliations:** 1 Department of Surgery (Surgical Laboratory), Academic Medical Center, University of Amsterdam, Meibergdreef 9, 1105 AZ Amsterdam, The Netherlands; 2 Department of Translational Physiology, Academic Medical Center, University of Amsterdam, Amsterdam, The Netherlands; 3 Cori-Tech, Bronkhorst High-Tech BV, Ruurlo, The Netherlands

**Keywords:** Normothermic, Hypothermic, Coriolis flow sensor, Ultrasonic flow sensor, Waveform

## Abstract

Intra-organ flow is the most critical parameter in machine-perfused organ preservation systems (MPS). Ultrasonic flow sensors (UFS) are commonly employed in MPS. However, UFS are sensitive to changes in fluid composition and temperature and require recalibration. Novel Coriolis-type mass flow sensors (CFS) may be more suitable for MPS because the measurement technique is not amenable to these factors. The effect of viscosity, colloids, temperature, pressure, and preservation solution on flow measurement accuracy of UFS and CFS was therefore investigated. A CFS-based MPS was built and validated for setpoint stability using porcine kidneys and the ability to reproduce different pressure and flow waveforms. The UFS exhibited a temperature- and preservation solution-dependent overestimation of flow rate compared to the CFS. The CFS deviated minimally from the actual flow rate and did not require recalibration. The CFS-based MPS conformed to the preprogrammed temperature, flow, pressure, and vascular resistance settings during 6-h kidney preservation. The system was also able to accurately reproduce different pressure and flow waveforms. Conclusively, CFS-based MPS are more suitable for organ preservation than UFS-based MPS. Our CFS-based MPS provides a versatile yet robust experimental platform for testing and validating different types of clinical and experimental MPS.

## Introduction

A chronic shortage of donor organs has been the driving force for an expansion of the donor pool with non-heartbeating donor (NHBD) organs.^13^ Due to their origin, these organs are subjected to extensive organ damage as opposed to organs retrieved from heartbeating or living donors. Despite the advantages of hypothermic (4 °C) organ preservation, the clinical standard in transplantation programs, hypothermia leads to post-preservation organ damage following transplantation and reperfusion due to intracellular energy-, metabolite-, and anti-oxidant shortage.^3^,^22^ As a consequence, renewed interest in *ex vivo* rewarming prior to transplantation and (sub)normothermic machine perfusion (32–37 °C) has arisen with the aim to prevent the detrimental effects associated with hypothermia.^5^,^12^,^19^–^21^

For use under (sub)normothermic conditions, preservation solutions must contain metabolism-supporting substrates, colloids to prevent edema, and ideally, oxygen carriers.^8^ The complexity of these solutions has implications for the dynamics of machine perfusion (i.e., pressure, flow, and waveform) that in turn, may affect accuracy of flow measurements in preservation solutions due to their temperature-dependent rheological properties.^2^,^18^

Perfusate flow remains the most commonly used parameter for the quality of organ perfusion. Accuracy of flow measurement, therefore, is imperative since flow and pressure are used to calculate intra-organ vascular resistance (VR, mmHg mL^−1^ min^−1^) that is regarded the main indicator of micro-vascular integrity.^4^,^6^,^9^,^17^

Ultrasonic-based sensors are usually applied in the experimental setting of hypo- or normothermic machine perfusion. Although they are easy-to-use and comprise a broad array of clamp-on tubing sensors, changes in perfusion temperature, pressure, and applied preservation solution can affect the accuracy of flow measurements.^8^,^10^ In addition, ultrasonic sensors require recalibration for temperature and solution changes, restricting their application in organ resuscitation by *ex vivo* rewarming prior to implantation. Using a Coriolis-based mass flow sensor that measures flow based on the solutions’ mass instead of volume or the Doppler effect may circumvent these drawbacks. Due to this property, experimental machine perfusion systems with Coriolis-based flow sensors might enable reproduction of clinically and experimentally encountered flow dynamics with a greater accuracy under changing preservation conditions.^1^

The aim of this study was to determine the accuracy of flow measurements using ultrasonic- and Coriolis-based flow sensors with respect to temperature-dependent viscosities and pressure variations of the following preservations solutions: University of Wisconsin (UW), histidine–tryptophan–ketoglutarate (HTK), and the experimental preservation solution, Polysol (PS). On the basis of these findings, an experimental machine perfusion system incorporating Coriolis-based mass flow technology was developed and validated for temperature, flow, and pressure stability with the final goal to provide reproducible generation of versatile flow patterns (waveforms). This development intended to provide a platform for optimal perfusion parameter determination.

## Materials and Methods

### Viscosity Measurements in Preservation Solutions

Temperature and preservation solution-dependent viscosity was measured using a LV DV-II + Pro Digital Rheometer (Brookfield Engineering Laboratories, Middleboro, MA) and Rheocalc 3.1-1 (Brookfield Engineering Laboratories) software for data acquisition. Samples of preservation solution (500 μL) were transferred to the measurement cup that was kept at 4, 15, 20, 28, or 37 °C using a water bath (Tamson Instruments, Zoetermeer, The Netherlands). Temperature-dependent viscosity (*N* = 7 for each solution) was determined in demineralized water (DW, Baxter, Deerfield, IL), histidine-tryptophan-ketoglutarate (HTK, Dr F Köhller-Chemie, Bensheim, Germany), Polysol (PS, Mediphenix, Amsterdam, The Netherlands), and University of Wisconsin solution (UW, Fresenius Hemocare, Emmer-Copascuum, The Netherlands). The characteristics of the solutions are provided in Table 1.

**Table 1:** Composition of solutions used

	HTK	UW	PS	RL	DW
Colloids (g L^−1^)					
PEG			20 (35 kDa)		
HES		50			
Impermeants (mmol L^−1^)					
Mannitol	38				
Lactobionate		100			
Raffinose		30	3.2		
Trehalose			5.3		
Sodium gluconate			75		
Potassium gluconate			20		
Buffers (mmol L^−1^)					
Histidine	198		6.3		
KH_2_PO_4_		25			
NaH_2_PO_4_			21.7		
HEPES			24		
Electrolytes (mmol L^−1^)					
Calcium	0.0015			1.5	<2
Chloride	32	20		109	<2
Magnesium	4				<2
Magnesium sulfate		5			
Potassium	9	120	15	4	<2
Sodium	15	25	120	130	<2
Anti-oxidants (mmol L^−1^)					
Tryptophan	2				
Allopurinol		1			
Glutathione		3	5.6		
Ascorbic acid			0.11		
Alpha-tocopherol			5.4 × 10^−5^		
Additives (mmol L^−1^)					
Ketoglutarate	1				
Adenosine		5	5		
Vitamins			^$^		
Amino acids			^$$^		
Lactate				28	
Osmolarity (mOsm L^−1^)	310	325	325	272	<1
pH	7.02–7.2	7.4	7.4	6.5	7

HTK: Histidine-tryptophan-ketoglutarate; UW: University of Wisconsin solution; PS: Polysol; RL: Ringers lactate; DW: Demineralized water; PEG: Polyethylene glycol; HES: Hydroxyethyl starch

^$^(mmol L^−1^): ascorbic acid (0.11), biotin (0.21), Ca-pantothenate (0.004), choline chloride (0.01), inositol (0.07), ergocalciferol (3 × 10^−4^), folic acid (0.002), menadione (4 × 10^−5^), nicotinamide (0.01), nicotinic acid (0.004), pyridoxal (0.005), riboflavin (0.003), thiamine (0.03), vitamin A (3 × 10^−4^), vitamin B12 (1 × 10^−4^), and vitamin E (5 × 10^−5^)

^$$^(mmol L^−1^): alanine (1.01), arginine (1.18), asparagine (0.08), aspartic acid (0.23), cysteine (0.33), glutamic acid (0.34), leucine (0.57), glutamine (0.002), glycine (0.67), isoleucine (0.38), lysine (0.48), methionine (0.30), ornithine (2.00), phenylalanine (0.30), proline (0.78), serine (0.29), threonine (0.34), tryptophan (0.88), tyrosine (0.19), and valine (0.43)

### Assessment of Flow Measurement Accuracy

Accuracy of flow measurement was determined in a custom-built setup in which two commercial systems of equal measurement capacity (250 mL min^−1^) were placed according to the manufacturers’ instructions, being an ultrasonic in-line sensor (ME4PXN, Transonic, Ithaca, NY on a TS410 console) and a Coriolis mass flow controller (Cori-Flow M14; Bronkhorst Cori-Tech BV, Ruurlo, the Netherlands). Flow was generated using a non-pulsatile gearpump (DGS.11; Tuthill, Alsip, IL) and allowed to stabilize at approximately 50 mL min^−1^. Pressure was controlled by a pressure transducer (El-Press P-502C-350R; Bronkhorst High-Tech, Ruurlo, the Netherlands) at 30, 55, 75, or 95 mmHg (zero stability has been determined by the manufacturer) and temperature was set to 4, 15, 20, or 28 °C using a water bath (Tamson Instruments). Flow measurement accuracy was related to the flow rate derived from a precision weighing scale (PR8002; Mettler-Toledo, Tiel, the Netherlands) that was continuously read-out by a custom-built program (Bronkhorst High-Tech, Ruurlo, the Netherlands). The program calculated the volumetric flow (mL min^−1^) reference by means of the weight increase and density (measured prior to each experiment) of each solution.

The ultrasonic sensor was recalibrated according to the manufacturers’ instructions prior to each change of solution and temperature, whereas the Coriolis sensor (manufactured and calibrated in 2009) did not require any recalibration according to its design. All hardware components were controlled by a FlowBus module (Bronkhorst High-Tech) and data were collected in FlowPlot (version 3.28, Bronkhorst).

### Experimental Machine Perfusion System

**Figure 1:**
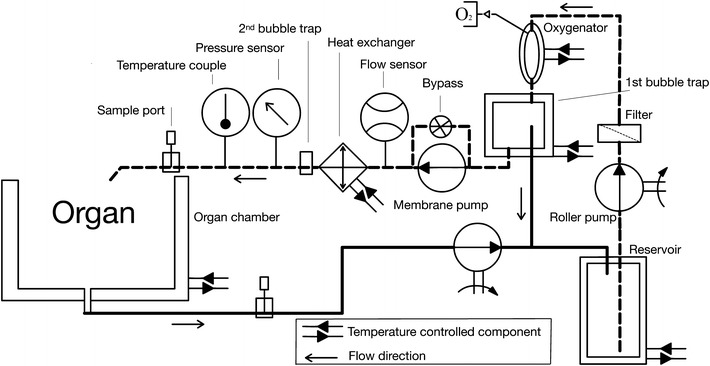
Schematic representation of the machine perfusion system. The perfusion solution in the reservoir passes a roller pump, filter, oxygenator, bubble trap, miniature membrane pump, mass flow transducer, heat exchanger, 2nd bubble trap, pressure sensor, and temperature couple prior to entering the organ’s artery

A schematic overview of our custom-engineered machine perfusion system is depicted in Fig. 1. Briefly, 1 L of perfusion solution was kept in a glass reservoir, oxygenated by a custom-built glass oxygenator (passive diffusion-based), and two custom-built glass bubble traps. Perfusate temperature was controlled by a custom-built glass heat exchanger. The perfusion system was driven by a rollerpump (Watson-Marlow, Wilmington, MA), pumping perfusion solution from the reservoir to the first bubble trap while a miniature membrane pump (NF60KPDCB-4, KNF Neuberger GMBH, Freiburg, Germany) provided the arterial perfusion pressure after the first bubble trap. The membrane pump was combined with two bypasses in order to provide non-pulsatile flow to the organ.

All glass components were temperature-controlled by a waterbath (HMT-200; HETO, Holten, the Netherlands) and thermocouple (C8.B Licox; Integra, Vilvoorde, Belgium). A pressure controller (El-Press P-502C-350R; Bronkhorst) was positioned directly prior to the glass organ chamber for pressure regulation. A gas flow controller (O_2_ El-Press F-201CV; Bronkhorst) coupled to a 50-L oxygen-containing cylinder (Hoekloos Medical, Amsterdam, the Netherlands) subsequently regulated oxygenation of the perfusate. All hardware components were connected to a data acquisition board (NI USB-6229 BNC, National Instruments, Austin, TX) and controlled by a custom-written program in Labview (version 8.4.2, National Instruments).

#### Stability of the Machine Perfusion System Settings

System setting stability was determined using a non-pulsatile flow of 50 mL min^−1^ in a pressure range of 0 to 95 mmHg and temperatures of 4, 15, 20, 28, or 37 °C. As perfusion solution, PS was used inasmuch as it was specifically designed as a colloidal machine perfusion solution. Stability was defined as the capability of the perfusion system to comply with the preprogrammed setpoint with respect to flow, pressure, and temperature. All measurements were performed over a period of 5 min and repeated five times in a random fashion within the same temperature group.

#### Stability During *Ex Vivo* Kidney Preservation

After approval of the animal ethics committee (BEX 102084) and in accordance with the principles of laboratory animal care, two non-heartbeating kidneys from two healthy, 50-kg female Landrace pigs were retrieved as previously described^14^ and connected to the preservation system. The kidneys were perfused for 6 h *via* the renal artery with free venous outflow. PS with 10% v/v PFOB emulsion^11^ as oxygen carrier was used as preservation solution at 28 °C and a fixed perfusion pressure of 75 mmHg. Temperature, pressure, and flow were continuously measured using the previously mentioned setup.

#### Multi-Functionality of the Experimental Machine Perfusion System

To confirm multi-functionality of the perfusion system, complex waveforms encompassing pressure spikes or smooth pressure control were selected to ascertain the ability to mimic commercial devices with different perfusion profiles. Although rapid pressure spikes are not clinically demanded, they provide insight in the capabilities of rapid adaptations by the perfusion setup. To this end, sinusoidal, sawtooth, and block waveforms were preprogrammed in Labview and sent as setpoints to the perfusion system. The waveforms’ characteristics were composed of a 10-mmHg offset and 10-mmHg amplitude as frequently used in the clinical setting, and the baseline flow was set at 10 mL min^−1^ to allow pressure release at a 0-mmHg setpoint.

While most commercial perfusion preservation devices generate a sinusoidal waveform, a theoretical, more complex waveform composed of fast and smooth pressure changes combined with a stabilization phase was created to mimic pre-clinical devices like the Airdrive.^15^ The waveform characteristics were defined as a 15-mmHg offset and 10-mmHg amplitude in a one-second timespan but with a baseline flow of 20 mL min^−1^ to allow pressure drops. Each waveform measurement was reproduced in tenfold in random order at 20 °C to determine reproducibility of waveform generation by the system.

### Statistical Analysis

Differences were statistically analyzed for significance using GraphPad Prism (GraphPad software, La Jolla, CA). For intra- and intergroup differences in the viscosity experiments, a Kruskal–Wallis test with *post hoc* Dunn’s test was employed. All other ordinal differences were analyzed by a two-way ANOVA with Bonferroni *post hoc* test. Flow measurement accuracy was displayed using a Bland–Altman plot using the comparison: difference (*A*–*B*) vs. average in which the difference is defined as the precision weighing scale result minus the Coriolis or ultrasound result, plotted against their averaged result. A *p*-value of <0.05 was considered statistically significant.

## Results

### Viscosity of Preservation Solutions

**Figure 2:**
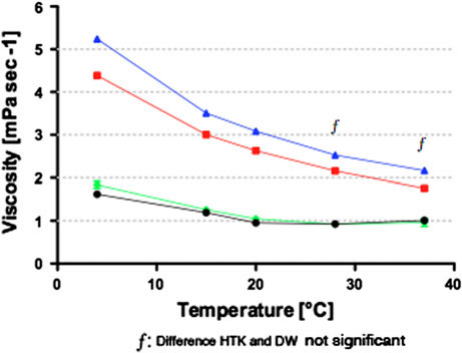
Viscosity of UW (blue), PS (red), HTK (green), and RL (black) showing a clear temperature-dependent effect

Colloidal solutions showed a significant temperature-dependent viscosity while non-colloidal solutions did not (Fig. 2). The viscosity of the colloidal solutions UW and PS was 2.5-fold higher at 4 °C compared to 37 °C (*P* < 0.0001). This effect was less pronounced for the non-colloidal solution HTK that exhibited a 1.6-fold difference (*P* < 0.0001). A significant difference between the viscosities of the solutions was found at all temperatures except for the non-colloidal solutions at 28 and 37 °C.

### Flow Measurement Accuracy

**Figure 3:**
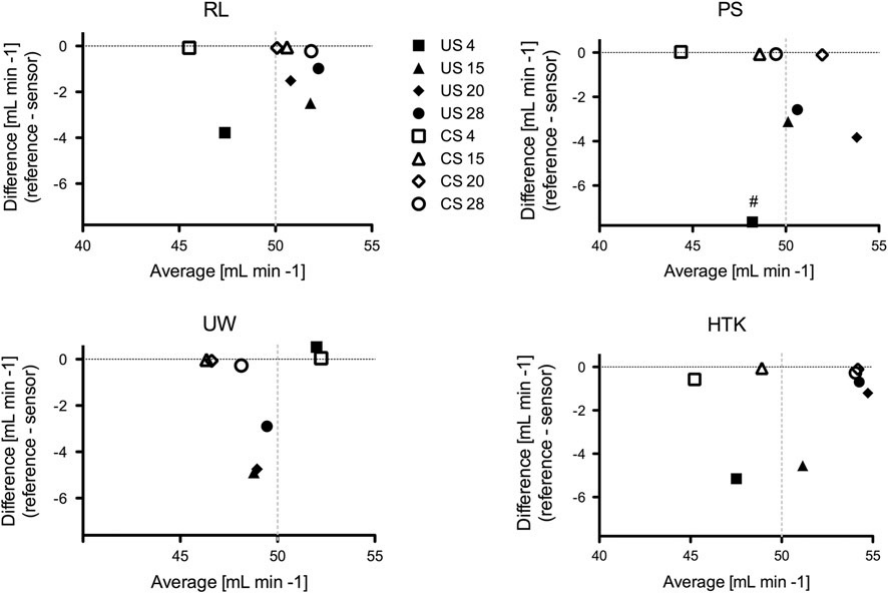
Bland–Altman plots [weighing scale—ultrasonic (US) or Coriolis (CS) vs. average] displaying overestimation of flow by the US technique in comparison to the CS technique

Flow measurement accuracy of the ultrasonic- and Coriolis-based sensors related to the precision weighing scale reference was mainly determined by the solution used, as evidenced by the Bland–Altman analysis (Fig. 3).

The Coriolis mass flow sensor showed very little-to-none deviation from the flow measurement reference, regardless of preservation solution type and temperature. The maximum flow rate overestimation by the Coriolis sensor was 0.5 mL min^−1^ in the 4 °C HTK group.

The ultrasonic sensor displayed a more pronounced overestimation of the flow rate at 4 and 15 °C, with a minimum of 3.7 mL min^−1^ for RL and a maximum of 7.6 mL min^−1^ for PS. However, a colloid-dependent effect could not be demonstrated at these temperatures as shown by the inter- and intra-group analyses (Table 2). At higher temperatures (20 and 28 °C) the ultrasonic flow sensor (UFS) displayed greater accuracy with non-colloidal solutions (HTK and RL) in comparison to colloidal solutions (UW and PS, Table 2).

**Table 2:** Statistical differences in measurement accuracy between solutions per temperature group

Temperature (°C)	Solutions	*p* value
Coriolis sensor		
20	PS vs. UW	0.014
Ultrasonic sensor		
4	PS vs. UW	<0.001
	PS vs. HTK	<0.001
	PS vs. RL	<0.001
15	PS vs. UW	<0.001
	PS vs. HTK	<0.001
	PS vs. RL	0.034
	UW vs. HTK	<0.001
	HTK vs. RL	<0.001
20	PS vs. HTK	<0.001
	PS vs. RL	0.001
	UW vs. HTK	<0.001
	UW vs. RL	<0.001
28	PS vs. HTK	<0.001
	PS vs. RL	<0.001
	UW vs. HTK	<0.001
	UW vs. RL	<0.001

HTK: Histidine–tryptophan–ketoglutarate; UW: University of Wisconsin solution; PS: Polysol; RL: Ringers lactate; DW: Demineralized water

Finally, the flow rate measurement accuracy of the ultrasonic and Coriolis flow sensor was not influenced by pressure variations, regardless of temperature and the preservation solution applied (data not shown).

### Stability of the Experimental Machine Perfusion System Settings

The perfusion system performed in excellent compliance with the pre-programmed setpoint (Table 3). With respect to flow stability, the measured standard deviation (SD) from the setpoint did not exceed 0.317 mL min^−1^ across the assessed temperature range of 4–37 °C. Regarding the perfusion pressure, the system could properly manage the pressure setpoint, indicated by the maximum pressure SD of 0.022 mmHg. This minimal SD from the pressure setpoint indicates the ability to provide non-pulsatile flow. Furthermore, at the 50-mL min^−1^ flow rate, the preservation solution temperature showed a maximum SD from the setpoint of 0.426 °C at 20 °C and even lower at the other temperatures.

**Table 3:** Machine perfusion system settings stability

Temperature (°C)	Setpoint	Mean	SD
Flow (mL min^−1^)			
4	50	49.950	0.098
15	50	50.062	0.257
20	50	49.891	0.170
28	50	49.881	0.125
37	50	50.036	0.317
Pressure (mmHg)			
	30	30.005	0.022
	55	55.000	0.002
	75	75.001	0.003
	95	94.999	0.003
Temperature (°C)			
	4	3.990	0.036
	15	14.819	0.411
	20	20.224	0.426
	28	28.040	0.120
	37	37.013	0.371

SD: standard deviation

**Figure 4:**
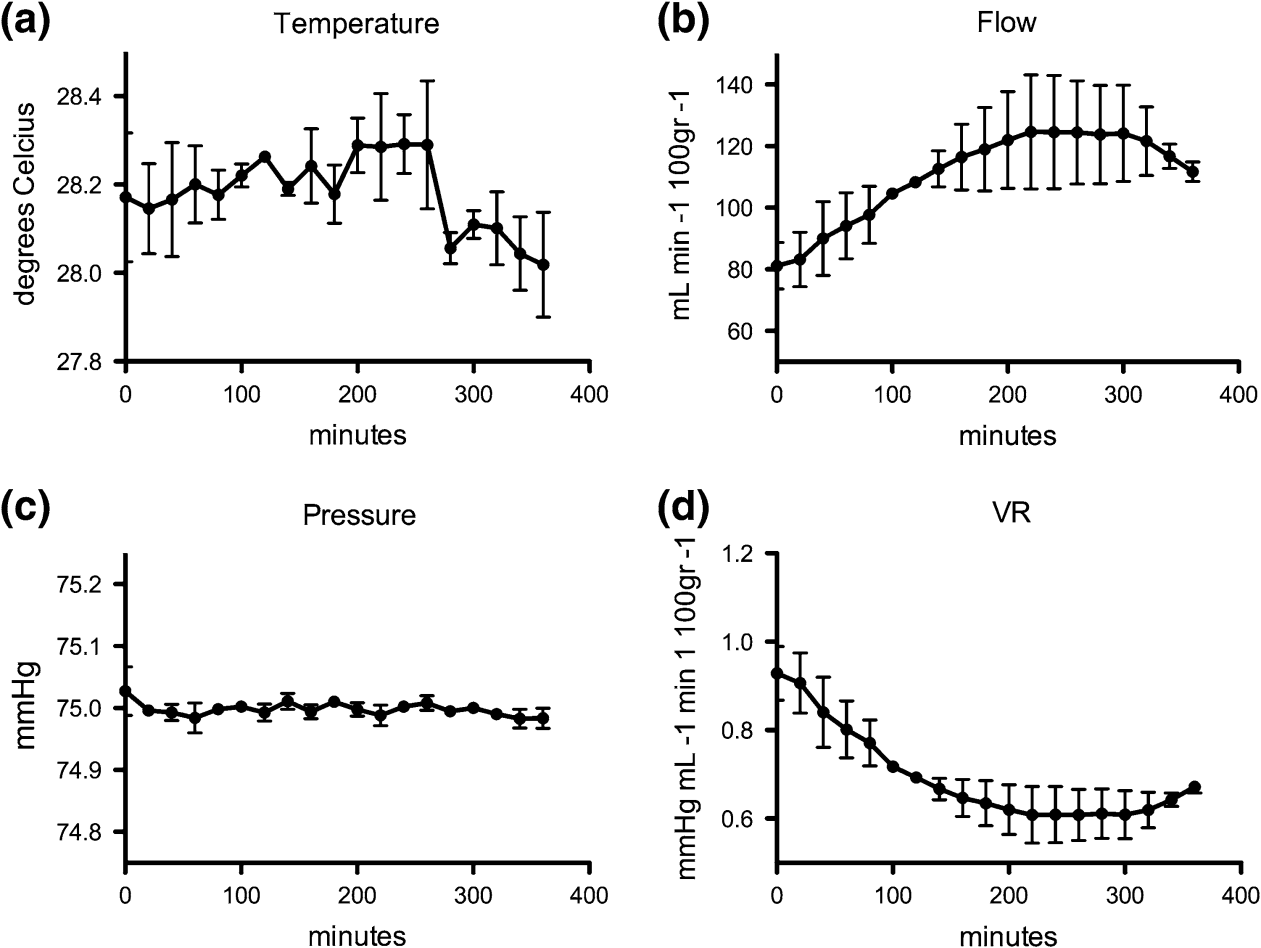
The dynamics of temperature (a), flow (b), pressure (c), and vascular resistance (VR, d) during 6 h *ex vivo* machine perfusion preservation of 2 NHBD porcine kidneys at 28 °C

*Ex vivo* perfusion of NHBD kidneys resulted in a stable preservation solution flow of 116 ± 9.06 mL min^−1^ 100 g^−1^ (mean ± SD) over the 6-h preservation period. In this time, the temperature was maintained at 28.25 ± 0.09 °C and perfusion pressure at 74.92 ± 0.33 mmHg. The results are displayed in Fig. 4.

### Multifunctionality of the Machine Perfusion System

**Figure 5:**
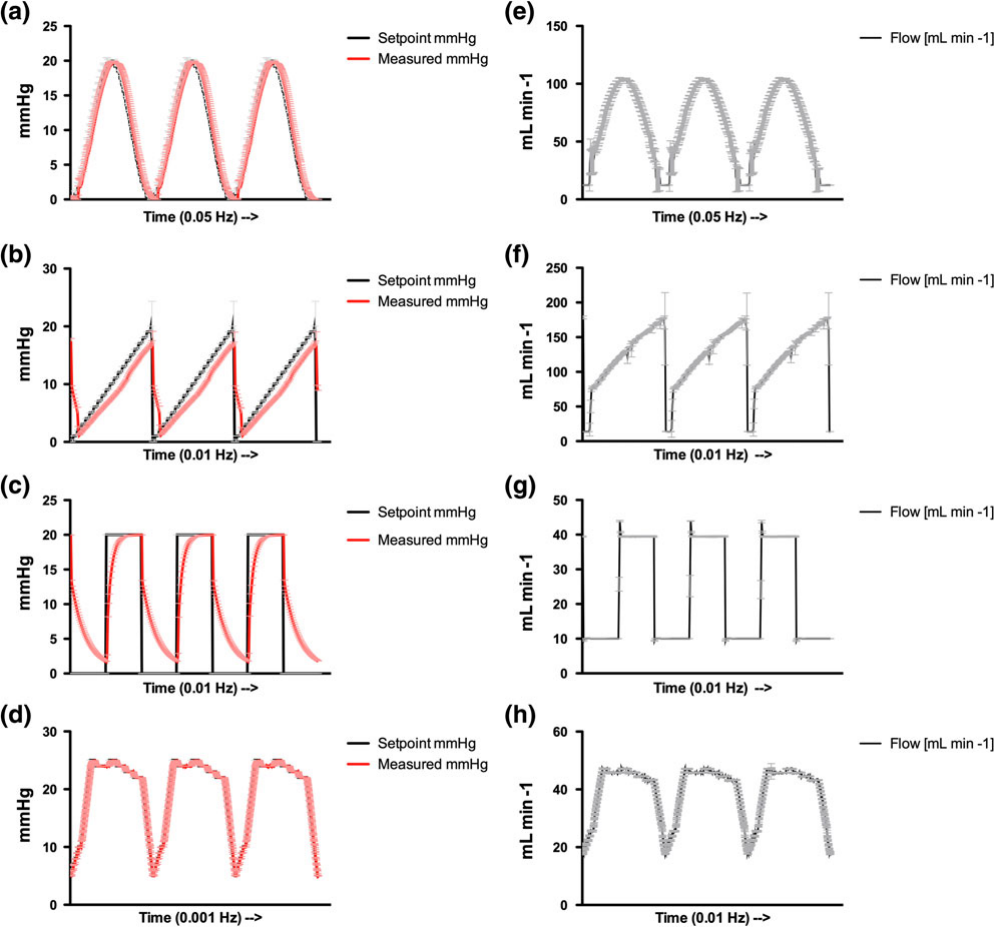
Waveform generation in the experimental system simulating a sinusoidal (a), sawtooth (b), block waveform (c), and complex composed waveform (d). The red line represents measured pressure (mmHg) and the black line represents the setpoint given to the controller device. (e)–(h) show the associated flow patterns (mL min^−1^)

Sinusoidal and more complex waveforms were generated in the machine perfusion system to ascertain the ability to reproduce clinical and experimental machine perfusion devices. In Fig. 5, a clear correlation is illustrated between the preprogrammed and measured waveform pressures and associated flow rates. The *R* ^2^ value for the sinusoidal, sawtooth, block, and composed waveforms were 0.987, 0.821, 0.829, and 0.997, respectively. The goodness of fit statistics confirms the system’s capability to preprogram and execute predefined waveforms, suggesting that, when properly defined, waveforms encountered in the clinical setting can be duplicated.

In this hardware setup, however, rapid pressure changes demand a slight time delay for pressure build-up and regulation by the pump and pressure controller. Without the clinical need for such rapid pressure changes, no hardware changes were applied in the setup. In support of the latter, a setpoint delay was not found in the corresponding flow waveforms and their *R* ^2^ values (0.983, 0.893, 0.954, and 0.998 for a sinus, sawtooth, block, and composed waveform, respectively). Therefore, perfusion was properly maintained despite a lag-phase in pressure control.

## Discussion

This study established that an inverse proportional relationship exists between preservation solution viscosity and temperature, an effect most pronounced in the colloid-containing solutions. Furthermore, temperature and type of preservation solution considerably affected flow measurement accuracy when using an ultrasonic-based flow sensor but not a Coriolis-based mass flow sensor. Incorporation of the Coriolis-based sensor in our experimental machine perfusion system proved to perform in compliance with preprogrammed flow, pressure, and temperature. Moreover, compliance with complex waveforms enabled the system to recreate well-defined flow patterns of commercially available and experimental machine preservation systems.

In experimental perfusion preservation systems the ultrasonic-based flow sensors are frequently applied and pose no risk for impaired measurement accuracy when the preservation solution and applied temperature are left unchanged. However, with the hypothermia-related detrimental effects on preserved organs during reperfusion, interest in the complex process of organ rewarming prior to reperfusion is increasing. For the application of (sub)normothermic preservation, the effects of oxygen-carrying particles, changing perfusion solutions, and perfusion temperatures must be taken into account.^7^ In this respect, changes in the solution’s composition impact the density and viscosity, leading to a change in conductivity of the ultrasonic wave used in ultrasonic-based sensors.^2^,^18^ Changes in viscosity, in turn, can cause velocity variations across the tubing diameter due to friction at the tubing walls, impairing the flow rate measurement accuracy in older generation sensors.^10^,^23^ More importantly, particles present in preservation solutions (microbubbles, protein aggregates, residual erythrocytes, artificial oxygen carriers, etc.) are constantly influencing the ultrasonic flow measurement during perfusion, which impacts its reliability.^2^

**Figure 6:**
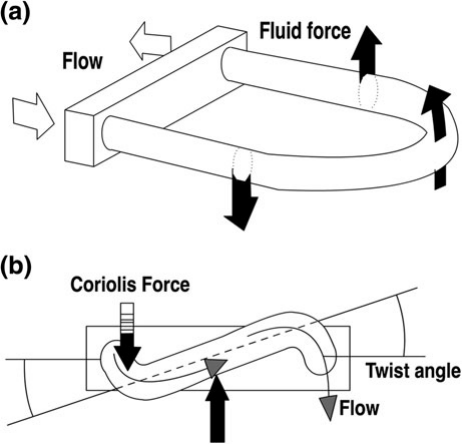
Principle of the Coriolis mass flow measurement, illustrating the forces and flow tube torsion when flow is present

**Figure 7:**
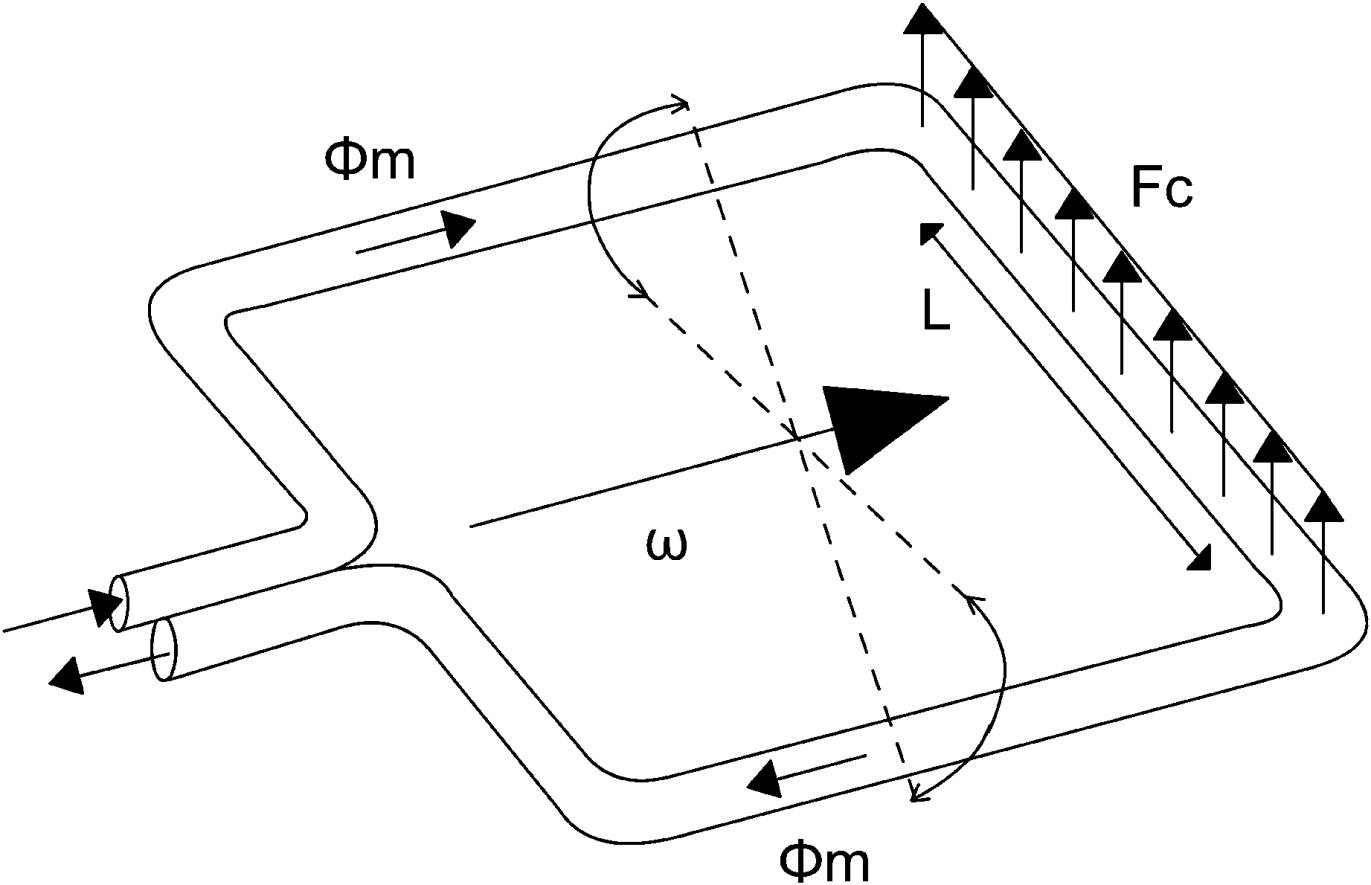
Coriolis flow sensor in which ω is the torsion mode actuation vector, *F*_c_ indicates the Coriolis force as a result of Φ_m_, being mass flow

The major advantage of Coriolis-based flow sensors over ultrasonic sensors is that their measurement is based upon mass flow instead of volume or velocity (Doppler). Coriolis-based sensors are therefore capable to correct for temperature, density, and particle-related changes of the solutions flowing through the sensor. In a Coriolis flow sensor, two U-shaped tubes are counter-vibrating within an 80- to 1000-Hz range, depending on the flow tube size. During flow, the vibration-amplitude at the inflow of the flow tube becomes ‘out-of-sync’ with the outflow amplitude due to the Coriolis force that moves the tube in a direction perpendicular to the flow and imposed rotational axis (Fig. 6). The Coriolis effect thereby facilitates the calculation of the exact mass flow from the phase-shift that occurs using the formula *F*_c_ = −2*L*ωΦ_m_ in which, *L* is the flow tube length, ω the torsion applied, and Φ_m_ the resulting mass flow (Fig. 7). Mass flow can subsequently be converted to volumetric flow by dividing it by the density of the fluid. Density is easily extrapolated because the given vibration frequency is dependent on the given mass of the flow tubes and the unknown density of the solution passing through the flow tubes.

The abovementioned corrections performed in a Coriolis-based flow sensor make the required recalibration prior to solution- or temperature-changes, which are obligatory for ultrasonic sensors, redundant. This is advantageous when normothermic organ resuscitation is applied, especially when taken into account that rewarming can take up to 68 min.^16^

The reproduction of various pressure and flow waveforms using the Coriolis technique with automatic, auto-feedback pressure regulation enabled the simulation of other experimental and commercial perfusion preservation devices. This allows research into optimal preservation conditions in one apparatus with inclusion of the current, clinically employed machine preservation devices. However, the application of Coriolis-based sensors for clinical organ preservation is currently not possible for technical reasons. The current layout of the sensor and especially the flow tube comprises frequent bends and dead volumes. With the strict clinical regulations and the inability to autoclave the sensor with the attached electronics, clinical sterility guidelines cannot be followed. Furthermore, as the sensor’s measurement principle is based on the distortion of a generated vibration, the coriolis-based measurement is sensitive to external vibrations. Without hardware changes to overcome these issues, implementation in a portable, clinically useable system is not feasible. Therefore, this flow measurement technique and perfusion system described in this work are as yet specifically aimed at the development of (sub)normothermic preservation techniques in the experimental setting in a non-portable setup.

In conclusion, flow sensors based on the Coriolis technique measure flow rate more accurately without the need for recalibration regardless of preservation solution or temperature employed during machine perfusion. The presented preservation system proved to provide accurate flow and pressure management throughout temperature and preservation solution changes. The systems’ stability was hereafter confirmed with the perfusion of NHBD kidneys, safeguarding extrapolation of our results to the setting of organ perfusion preservation. Moreover, the system could comply with preprogrammed flow- and pressure-waveforms, facilitating the reproduction of defined flow and pressure waveforms of experimental and clinical machine perfusion devices. This project therefore resulted in a system capable of looking into optimal machine perfusion settings in a way that flow and pressure waveforms might be pre-validated.

## Abbreviations

NHBD: Non-heartbeating donor
HMP: Hypothermic machine perfusion (4 °C)
SCS: Static cold storage (4 °C)
NMP: Normothermic machine perfusion (37 °C)
VR: Vascular resistance (mmHg mL^−1^ min^−1^)
DW: Demineralized water
HTK: Histidine–tryptophan–ketoglutarate
PS: Polysol
UW: University of Wisconsin solution
RL: Ringer lactate
*F*_c_: Coriolis force (N m^−1^ s^−1^)
*L*: Length (m)
Ω: Greek, small letter Omega, torsion (degrees angulation)
Φ_m_: Greek, capital letter Phi, mass flow (kg h^−1^)
